# Supplementary material for: Predatory functional response and fitness parameters of Orius strigicollis Poppius when fed Bemisia tabaci and Trialeurodes vaporariorum as determined by age-stage, two-sex life table
Source: PeerJ. 2020 Jul 28;8:e9540. doi: 10.7717/peerj.9540 (PMC7394059; doi:10.7717/peerj.9540)

**Probability plot of RESl1 for Adult *O.* *strigicollis*** **fed On *B. tabaci* nymphs.**


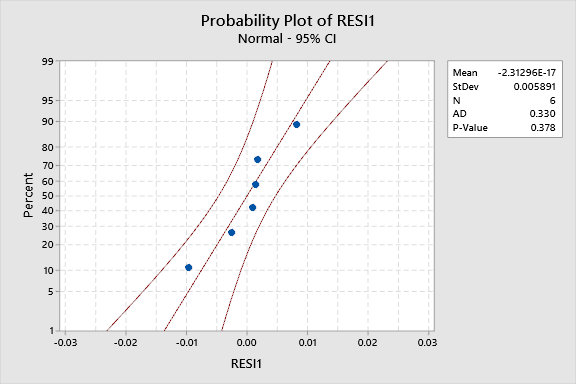


**
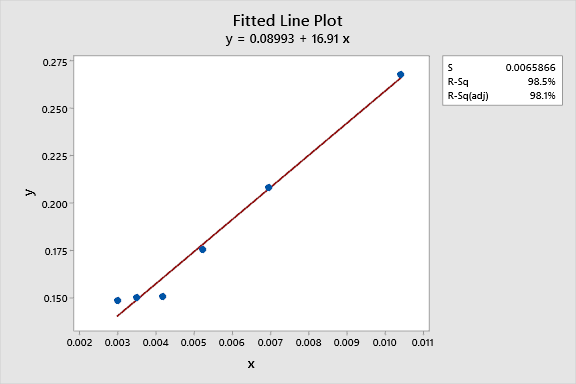
Fitted Line Plot for Adult *O.* *strigicollis*** **fed On *B. tabaci* nymphs.**

**Normal Probability plots for Adult *O.* *strigicollis*** **fed On *B. tabaci* nymphs.**


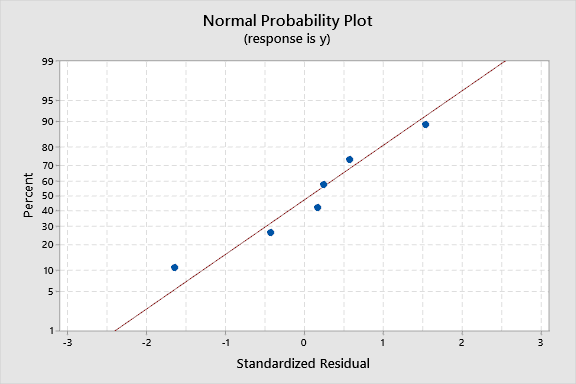


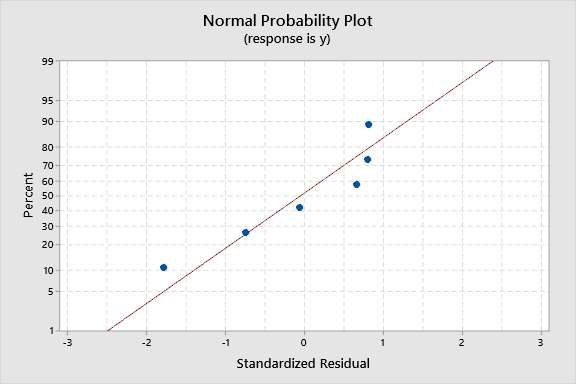
**Normal Probability plots for 3^rd^ instar *O.* *strigicollis*** **fed On *B. tabaci* nymphs.**

**Fitted Line plots for 3^rd^ instar *O.* *strigicollis*** **fed On *B. tabaci* nymphs.**

**
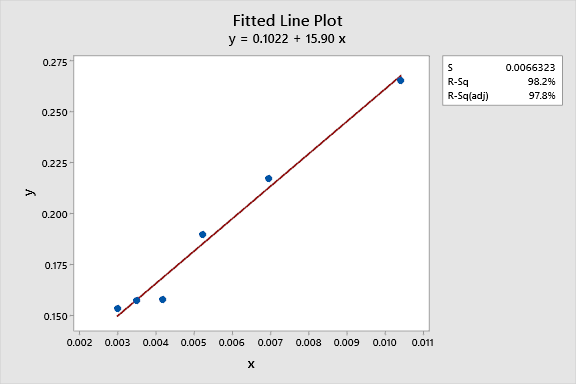
**

**Probability plot of RESl1 for 3^rd^ instar *O.* *strigicollis*** **fed On *B. tabaci* nymphs.**


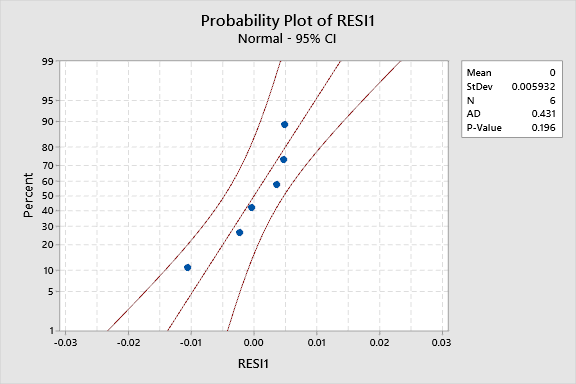


**Normal Probability plot for Adult *O.* *strigicollis*** **fed On *T. vaporariorum* nymphs.**
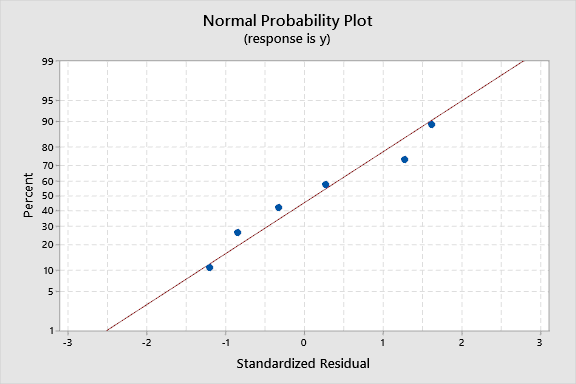


**
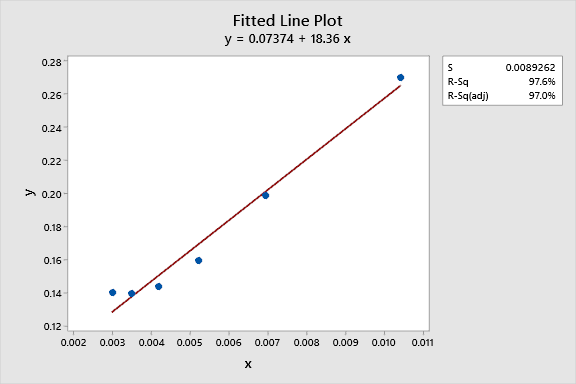
Fitted Line plots for Adult *O.* *strigicollis*** **fed On *T. vaporariorum* nymphs.**


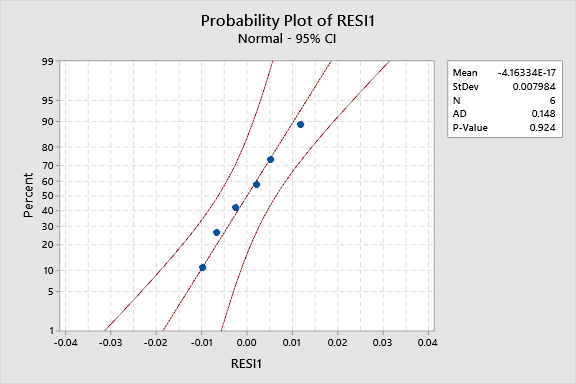
**Probability Plot of RESl1 for Adult *O.* *strigicollis*** **fed On *T. vaporariorum* nymphs**

**Normal Probability plot for 3^rd^ instar *O.* *strigicollis*** **fed On *T. vaporariorum* nymphs.**


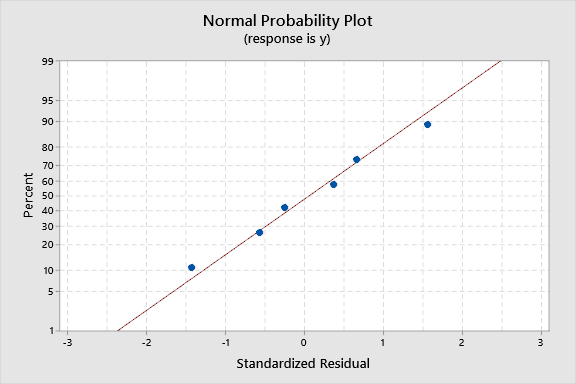


**Fitted Line plot for 3^rd^ instar *O.* *strigicollis*** **fed On *T. vaporariorum* nymphs.**


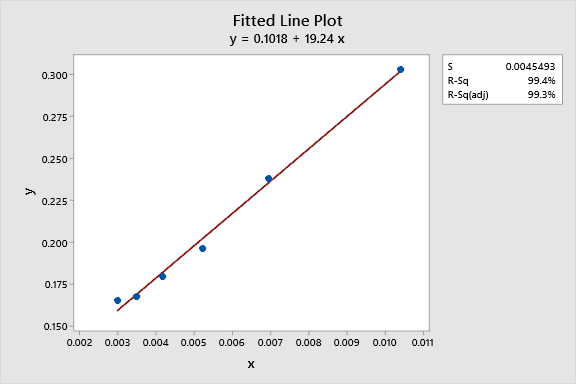


**Probability Plot of SRES1 for 3^rd^ instar *O.* *strigicollis*** **fed On *T. vaporariorum* nymphs.**


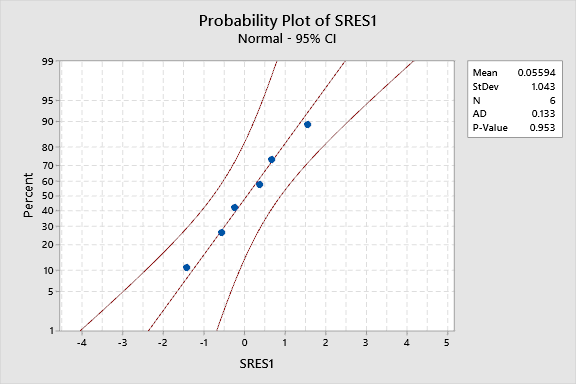

Supplement: Supplemental Information 1 [file peerj-08-9540-s001.docx]
